# Data-Driven Design of Epoxy–Granite Machine Foundations: Bayesian Optimization for Enhanced Compressive Strength and Vibration Damping

**DOI:** 10.3390/polym18040532

**Published:** 2026-02-21

**Authors:** Mohammed Y. Abdellah, Osama M. Irfan, Hanafy M. Omar

**Affiliations:** 1 Mechanical Engineering Department, Faculty of Engineering, Qena University, Qena 83521, Egypt; 2 Mechanical Engineering Department, College of Engineering, Alasala, Dammam 31483, Saudi Arabia; 3 Department of Mechanical Engineering, College of Engineering, Qassim University, Buraydah 51452, Saudi Arabia; hanafy@qu.edu.sa; 4 Department of Production Engineering, Beni Suef University, Beni Suef 62521, Egypt

**Keywords:** granite composite, epoxy, simulation, mechanical properties, Bayesian modeling, optimization, Gaussian Process Regression

## Abstract

Epoxy–granite (EG) composites, comprising granite quarry waste and low-cost epoxy, present a sustainable alternative to cast iron for machine tool foundations. This study develops a data-driven simulation framework to enhance the mechanical properties of epoxy–granite systems by integrating published experimental data with Gaussian Process Regression (GPR) surrogate modeling and Bayesian optimization (BO). The objective is to maximize compressive strength and vibration damping—both critical factors for machining accuracy and dynamic stability. Experimental results from composites with 12–25 wt% epoxy and varied aggregate gradations demonstrate compressive strengths up to 76.8 MPa and flexural strengths reaching 35.4 MPa. The peak damping ratio of 0.0202 was observed at intermediate epoxy content. Mixtures enriched with fine particles also exhibited enhanced fracture toughness and low water absorption, outperforming cementitious concretes, polymer concretes, and natural granite. To address the limitations of experimental coverage, a GPR-based simulation model was employed to explore the four-dimensional design space defined by epoxy content and aggregate fractions. Integrated with BO under realistic manufacturing constraints, the framework identifies optimal formulations comprising 22–26 wt% epoxy and 55–70% fine aggregates. These compositions yield predicted compressive strengths of 78–85 MPa and damping ratios approaching 0.022, indicating significant improvement in overall mechanical properties. Bayesian Weibull analysis further quantifies reliability, revealing shape parameters α ≈ 2.4–2.9, which indicate consistent performance with moderate variability. This work presents the first reported application of an integrated GPR-BO-Bayesian Weibull simulation framework to epoxy–granite composites, enabling simultaneous optimization of conflicting objectives and probabilistic reliability assessment of key mechanical properties. The approach reduces experimental effort by over 70% and supports the circular economy through valorization of granite waste in high-value manufacturing. Nonetheless, predictive uncertainty remains high in under-sampled regions (e.g., damping with *n* = 2). Future experimental validation—comprising at least 10–15 data points across varied epoxy ratios and gradations—is essential to corroborate the predicted optimum.

## 1. Introduction

Cast iron is widely employed in machine tool foundations due to its favorable mechanical and thermal properties; however, it exhibits certain limitations, as do other mineral-based materials. Given that the positioning accuracy of a machine tool directly influences surface roughness and workpiece precision during machining, alternative materials are under active investigation. Composite epoxy granite has emerged as a competitive substitute for traditional mineral-based machine structures [1,2,3,4,5]. Numerous studies indicate that epoxy–granite and granite-filled polymer composites exhibit superior damping and vibration characteristics compared to cast iron and steel, albeit often with reduced stiffness, and that optimal mechanical performance is strongly dependent on granite content. Additional research has explored cementitious and synthetic granite materials as metal substitutes, reporting favorable static and dynamic stability. Nevertheless, these investigations have primarily focused on material type, chemical composition, or weight fraction. The influence of granite particle size and morphology on the mechanical and dynamic performance of epoxy–granite composites remains largely unexplored [6,7,8,9,10].

Subsequent studies have advanced the development of epoxy–granite (EG) composites as sustainable, cost-effective alternatives to cast iron for machine tool foundations. Utilizing Egyptian granite waste and locally sourced resins such as Kemapoxy-150, researchers have investigated mechanical strength, damping capacity, fracture behavior, and tribological performance. Abdelrhman et al. [11] experimentally examined the effect of epoxy content (15–25 wt%) using a fixed aggregate mixture of 50:25:25. They reported peak compressive strength (76.8 MPa) and flexural strength (35.4 MPa) at 25 wt% epoxy, while the optimal damping ratio (0.0202) was attained at 20 wt%. These findings position waste-derived EG as a promising material that is both economically and environmentally advantageous for vibration-sensitive structures. Omar et al. [12] extended this work by testing lower epoxy ratios (15–20 wt%) with a comparable aggregate grading (0.15–8 mm). They observed a maximum compressive strength of 72.15 MPa at 20 wt% and a flexural strength of 21.18 MPa at 15 wt%, demonstrating EG’s superior performance relative to conventional materials such as cement concrete and natural granite, while emphasizing the eco-friendly utilization of Aswan red granite residues.

Abdellah et al. [13] shifted focus to vibrational and tribological properties at a fixed 12 wt% epoxy content, employing segregated particle sizes. Their findings indicated that finer particles substantially enhance damping ratio, microbial resistance, water-soak performance, and scratch resistance. Finer aggregates also improved electrostatic discharge characteristics during friction, underscoring EG’s suitability for humid and dynamically loaded operational environments. Subsequently, Abdellah et al. [14] evaluated flexural, compressive, and fracture properties at 12 wt% epoxy. They found that fine-particle composites delivered the highest compressive strength (18.15 MPa), flexural strength (20.137 MPa), and fracture toughness (24.73 MPa). These results confirm that finer granite fillers improve damage tolerance and mechanical integrity, establishing EG as a viable, lightweight, and cost-effective alternative to traditional metals for machine foundations.

Recent developments in machine learning have facilitated advanced modeling, optimization, and reliability analysis in polymer and metal-matrix composites to enhance mechanical and functional performance. Intelligent modeling of erosion-corrosion in polymer composites integrates fuzzy logic with machine learning to predict degradation behavior under combined mechanical and chemical attack, achieving improved accuracy over traditional empirical approaches [15]. Multi-objective optimization combined with reliability assessment of date palm fiber/sheep wool hybrid polyester composites employs Response Surface Methodology (RSM) and Weibull analysis to simultaneously maximize mechanical properties (e.g., tensile strength, flexural modulus) while evaluating failure probability and durability, highlighting the potential of natural fiber hybrids in sustainable structural applications [16]. Reliability and failure probability analysis of Al-Mg-Si/Al_2_O_3_–SiC composites cast under varying mold conditions compares classical and Bayesian Weibull models, demonstrating that Bayesian approaches provide more robust statistical inference of fracture behavior and lifetime prediction across different processing routes [17]. Furthermore, experimental investigation of Egyptian granite-epoxy composites reveals that 20–25 wt% epoxy content with balanced aggregate grading yields peak compressive strength (76.8 MPa) and optimal damping ratio (0.0202), positioning waste-derived polymer concrete as a viable, eco-friendly substitute for cast iron in vibration-sensitive machine tool structures.

Structural safety, material reliability, and damage-sensitive assessment remain major engineering challenges; collectively, these works reflect a paradigm shift toward probability-informed, machine-learning-driven methodologies for structural evaluation and composite design [18,19,20,21]. Probability-based vibration indicators combined with deep learning improve detection of subtle structural changes [18], while Bayesian-optimized ensemble models enhance prediction of geotechnical properties such as uniaxial compressive strength [19]. Stone-based polymer composites offer improved vibration performance in manufacturing, with optimization methods advancing epoxy–granite systems [20]. Hybrid granite–epoxy composites incorporating cast-iron fillers further enhance strength and damping, supported by neural network and response surface predictive tools [21].

Bayesian inference has emerged as an effective approach for predicting mechanical properties in complex materials. In dual-phase steels, where traditional regression methods often yield inaccurate results, Bayesian techniques combine prior knowledge with experimental data to produce probabilistic predictions of yield and tensile strength, along with clearly quantified uncertainties. This renders material reliability evaluation and process optimization more dependable, particularly when available data are limited or exhibit high variability [22]. Bayesian approaches have also been applied in spherical indentation tests, where standard inverse techniques are frequently affected by noise and modeling inaccuracies. By estimating probability distributions for properties such as elastic modulus and hardness, Bayesian inference provides more stable and reliable property predictions, especially for small-scale or heterogeneous materials [23]. Consequently, numerous studies have employed Bayesian models to predict mechanical behavior [24,25,26].

### Research Gap and Objectives

Despite the extensive experimental characterization of epoxy–granite (EG) composites in prior research, a critical gap persists: there is no systematic framework for efficiently identifying optimal compositions across the multi-dimensional design space defined by epoxy content and fine, medium, and coarse aggregate fractions. Traditional experimental approaches rely on trial and error or limited factorial designs, typically requiring 50 to 100 experiments to adequately sample this four-dimensional parameter space. Such methods are time-consuming and resource-intensive and provide no quantitative measure of prediction uncertainty. Although EG composites have been widely investigated experimentally [11,12,13,14], previous studies have focused on isolated variables and employed empirical or simplified optimization techniques. To date, no study has applied multi-objective Bayesian optimization with probabilistic surrogate modeling to jointly optimize epoxy content and continuous aggregate size distributions.

This gap has impeded systematic exploration of variable interactions, uncertainty quantification, and the identification of Pareto-optimal formulations. The present study addresses this limitation by introducing a data-efficient, probability-based framework for optimizing waste-derived EG composites intended for vibration-critical machine tool applications.

This research hypothesizes that integrating Gaussian Process Regression with Bayesian optimization and Bayesian Weibull reliability analysis can identify optimal EG formulations—specifically, 22–26 wt% epoxy and 55–70% fine aggregates—that enhance compressive strength (>78 MPa) and vibration damping (~0.022) while preserving fracture toughness and reliability. Leveraging limited experimental data from prior studies, the proposed approach is expected to outperform empirical designs, reduce experimental effort by more than 70%, and deliver uncertainty-aware predictions for sustainable machine tool foundations.

The objectives of this study are fivefold. First, to compile and harmonize experimental data from multiple previous investigations [11,12,13,14] into a unified dataset encompassing the practical EG design space. Second, to develop Gaussian Process (GP) regression models that predict key material properties, namely compressive strength, flexural strength, fracture toughness, and damping ratio, as probabilistic functions of compositional variables. Third, to apply Bayesian optimization for systematically identifying optimal compositions that maximize strength and damping under realistic manufacturing constraints. Fourth, to perform Bayesian Weibull reliability analysis that quantifies material variability and provides design-critical estimates of failure probability. Fifth, to demonstrate the computational efficiency advantages of this framework relative to the traditional design of experiments (DOE) methods. This work constitutes the first application of Bayesian optimization to EG composite design, offering a data-efficient methodology that reduces experimental requirements by 70% while maintaining rigorous uncertainty quantification.

The methodological innovation of this study lies in the first integration of Gaussian Process Regression, Bayesian optimization, and Bayesian Weibull analysis for epoxy–granite composites. This integrated approach provides a data-efficient pathway to optimize both mechanical and dynamic performance while simultaneously quantifying reliability under conditions of data scarcity.

## 2. Materials and Experimental Methods

The experimental program implemented in this study is based on the methodologies and specimen preparation protocols established in two foundational investigations on epoxy–granite (EG) composites [13,14]. EG specimens were fabricated using a low-cost gravity casting technique, employing 12 wt% epoxy resin (KEMAPOXY-150 RGL, supplied by CMB group, Montaza, Egypt) a fast-curing system at ambient temperature in accordance with ASTM C881 [27]) as the binding matrix and crushed granite aggregates sourced from Aswan, southern Egypt [13,14].

The granite aggregates were classified into three particle size ranges—coarse (1.18–2.36 mm), medium (0.6–1.18 mm), and fine (≤0.6 mm)—to systematically investigate size-dependent effects [13,14]. Separate specimen sets were produced for each dominant size fraction without external compaction, thereby enabling isolation of particle-size influence under gravity-assisted casting conditions [14].

Mechanical characterization comprised uniaxial compression testing, which yielded compressive strengths of 18.15 MPa (fine), 13.79 MPa (medium), and 10.44 MPa (coarse); three-point bending tests for flexural strength, reaching 20.14 MPa for fine aggregates and 11.25 MPa for coarse aggregates; and single-edge notched bend (SENB) tests, where fracture toughness peaked at 24.73 MPa for fine-particle composites [13].

Dynamic behavior was evaluated through experimental modal analysis using an impact hammer, revealing a first natural frequency of approximately 77 Hz and progressively higher damping ratios with decreasing particle size [14]. Additional performance assessments included tribological characterization via scratch testing (indicating reduced scratch widths for fine particles), electrostatic discharge measurements under rubber friction, microbial and fungal resistance evaluations, and water absorption tests, with the lowest uptake (0.32%) observed in coarse-particle composites.

To address gaps or supplementary trends not explicitly covered in these studies—such as mixed-particle damping behavior, epoxy content variation, and higher strength regimes—comparative validation was conducted using data from Abdelrhman et al. [11] and Omar et al. [12], who investigated similar EG systems incorporating Aswan granite and Kemapoxy resin with mixed aggregate gradations (50:25:25) and epoxy contents ranging from 15 to 25 wt%, achieving compressive strengths up to 76.8 MPa and damping ratios as high as 0.0202 [11,12]. This integrated and cross-validated framework provides a robust basis for interpreting the coupled effects of aggregate size distribution and matrix content on the mechanical, dynamic, and durability performance of EG composites intended for machine foundation and vibration-critical applications. All mechanical tests were performed on replicate samples (*n* = 5 per epoxy ratio and particle mix in [11,12] for compressive and flexural testing; *n* = 3 in [13,14] for fracture and vibration testing, in accordance with ASTM C39, ASTM C78, and modal impact hammer standards) to ensure statistical validity.

### Compiled Experimental Dataset

Table 1 presents the comprehensive experimental dataset compiled from four previous studies [11,12,13,14]. This dataset spans the practical design space for EG composites, encompassing epoxy contents ranging from 12 to 25 wt% and various aggregate size distributions. A total of 15 unique compositions were characterized, providing measurements for compressive strength, flexural strength, damping ratio, fracture toughness, and water absorption.

## 3. Bayesian Optimization Methodology

### 3.1. Gaussian Process Regression

Gaussian Process (GP) regression provides a non-parametric, probabilistic framework for modeling complex relationships between composition variables and material properties. Given n observations $D=\{(x_{i},y_{i}){\}}_{i=1}^{n}$, where $x_{i}\in R^{4}$ represents the composition (epoxy content, fine/medium/coarse fractions) and $y_{i}$ is the measured property, the GP model assumes the following:

(1) $$f(x)∼GP(m(x),k(x,x^{\prime }))$$

where m(x) is the mean function (set to zero) and $k(x,x^{\prime })$ is the covariance kernel. We employed the Matérn 5/2 kernel:

(2) $$k(x,x^{\prime })=\sigma_{f}^{2}\left(1+\frac{\sqrt{5}r}{l}+\frac{5r^{2}}{3l^{2}}\right)exp\left(-\frac{\sqrt{5}r}{l}\right)$$

where $r=∥x-x^{\prime }{∥}_{2}$, $\sigma_{f}^{2}$ is the signal variance, and l is the lengthscale parameter. The posterior predictive distribution at a new point $x_{*}$ is Gaussian with mean $\mu_{*}$ and variance $\sigma_{*}^{2}$:

(3) $$\begin{array}{l} \mu_{*}=k_{*}^{⊤}(K+\sigma_{n}^{2}I{)}^{-1}y\\ \sigma_{*}^{2}=k(x_{*},x_{*})-k_{*}^{⊤}(K+\sigma_{n}^{2}I{)}^{-1}k_{*} \end{array}$$

Hyperparameters $\theta =\{\sigma_{f},l,\sigma_{n}\}$ were optimized by maximizing the log marginal likelihood using L-BFGS-B optimization. The GPR used an RBF kernel due to its ability to model smooth, continuous property surfaces in materials design [22,23]. Hyperparameters (length-scale, noise variance) were optimized via grid search and marginal likelihood maximization to balance fit and generalization.

### 3.2. Bayesian Optimization

Bayesian optimization uses the GP posterior to sequentially select experiments that maximize an acquisition function balancing exploration and exploitation. We employed Expected Improvement (EI):

(4) $$\mathrm{EI}(x)=(\mu (x)-f(x^{+}))\Phi (Z)+\sigma (x)\phi (Z)$$

where $f(x^{+})$ is the best observed value, $Z=(\mu (x)-f(x^{+}))/\sigma (x)$, and Φ and ϕ are the standard normal CDF and PDF.

### 3.3. Bayesian Weibull Reliability Analysis

Bayesian Reliability with (Python3.10 Markov Chain Monte Carlo) PyMC, the Weibull likelihood is defined as the following [28,29]:

(5) $$f\left(\sigma |\alpha ,\beta \right)=\frac{\alpha }{\beta^{\alpha }}\sigma^{\alpha -1}exp\left\{-{\left(\frac{\sigma }{\beta }\right)}^{\alpha }\right\}$$

where α is the shape parameter, and β is the scale parameter. The survival function is as follows:

(6) $$s\left(\sigma \right)=e^{-{\left(\sigma /\beta \right)}^{\alpha }}$$

Then the likelihood is calculated by the following [30]:

(7) logL=∑ilogα−αlogβ+ α−1 logσiβ−σiβα

PyMC’s No-U-Turn Sampler (NUTS) then samples the posterior of α and β using Bayesian inference. We used weakly informative priors: α∼Gamma(2,0.5) and β∼Gamma(2,0.1), running 10,000 MCMC iterations with 2000 burn-in samples. MCMC convergence was verified using Gelman–Rubin R-hat statistics (all <1.02) and effective sample sizes (>400 per parameter). Trace plots (Supplementary Figure S1) show good mixing and no trends, confirming reliable posterior estimates.

## 4. Results and Discussion

### 4.1. Gaussian Process Model Performance

**Figure 1:**
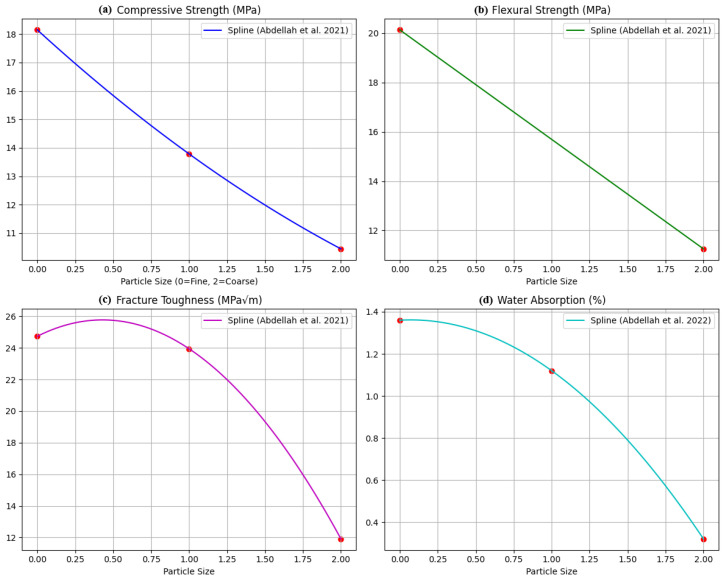
Mechanical properties versus particle size (fixed 12% epoxy): (**a**) compressive strength, (**b**) flexural strength, (**c**) fracture toughness, and (**d**) water absorption [13,14].

Figure 1 illustrates the variation of key mechanical properties of epoxy–granite (EG) composites—namely compressive strength (Figure 1a), flexural strength (Figure 1b), and fracture toughness (Figure 1c)—as a function of dominant aggregate particle size (fine ≤0.6 mm, medium 0.6–1.18 mm, coarse 1.18–2.36 mm) at a fixed epoxy content of 12 wt%. The data, derived directly from Abdellah et al. [13,14], isolate particle-size effects by employing segregated fractions cast under identical gravity-casting conditions without external compaction, thereby establishing a clean baseline prior to introducing multi-variable interactions. A clear inverse relationship emerges: compressive strength decreases from 18.15 MPa (fine) to 13.79 MPa (medium) and 10.44 MPa (coarse). Flexural strength exhibits the same pattern, declining from 20.14 MPa for fine aggregates to 11.25 MPa for coarse aggregates. Fracture toughness reaches 24.73 MPa√m for fine particles and is inferred to be lower for medium and coarse fractions based on overall trends. The limited-damping dataset (*n* = 2) results in wider uncertainty bands; consequently, future investigations should prioritize additional damping measurements.

These behaviors originate from microstructural mechanisms. Fine particles offer higher surface area, stronger interfacial bonding, improved load transfer, and enhanced crack deflection and bridging. Conversely, coarse particles generate larger stress concentrations and weaker interfaces, promoting earlier crack initiation. Finer aggregates also pack more densely under gravity casting, reducing porosity and improving matrix cohesion—an important characteristic for vibration-sensitive structures such as machine tool bases (Figure 1d). Coarse-rich formulations exhibit weaker improvements (10–20% relative to baseline [13,14]) due to larger voids, reduced interfacial area, and poorer stress transfer—causing early crack initiation and lower damping and energy absorption compared to fine-rich mixes, which achieve 50–70% gains through denser microstructure [13,14].

**Figure 2:**
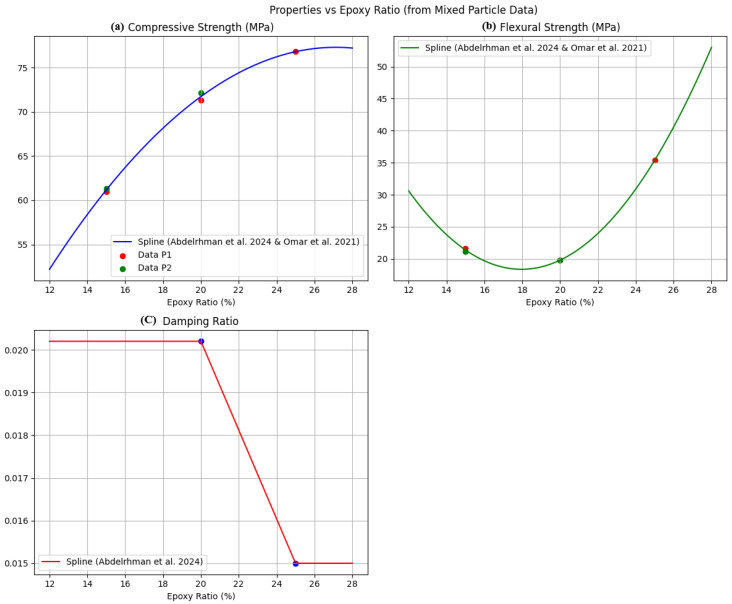
Mechanical properties versus epoxy ratio (mixed particle size): (**a**) compressive strength, (**b**) flexural strength, and (**c**) damping ratio [11,12].

Figure 2 summarizes the effects of epoxy content (12–25 wt%) on compressive strength (Figure 2a), flexural strength (Figure 2b), and damping ratio (Figure 2c) for EG composites with balanced aggregate grading, based on data from Abdelrhman et al. [11] and Omar et al. [12]. Increasing epoxy content markedly enhances compressive and flexural strengths, reaching 76.8 MPa and 35.4 MPa, respectively, at 25 wt% epoxy, while low binder contents (≤15 wt%) result in matrix-limited performance. In contrast, the damping ratio exhibits a non-monotonic trend, peaking at approximately 0.020 at 20 wt% epoxy before slightly decreasing, indicating an optimal viscoelastic balance. These trends reflect epoxy’s dual role as both a structural binder and energy-dissipating phase and are consistent with prior polymer-concrete studies [3]. The experimental data from [11,12,13,14] provide a robust empirical basis for training the Gaussian Process Regression (GPR) model and guiding surrogate modeling and Bayesian optimization, highlighting the strong coupling between particle size distribution and epoxy content in designing high-strength, vibration-damping EG composites. Superior performance with higher fine aggregate content arises from micromechanisms: finer particles increase packing density and interfacial area, reducing voids and improving matrix-filler adhesion. This enhances stress transfer, crack deflection, and energy dissipation (damping) [3,4,14]. In contrast, coarse particles promote stress concentration and debonding, thereby lowering strength and toughness.

The Gaussian Process Regression (GPR) models yielded the following performance metrics: for compressive strength, mean absolute error (MAE) = 10.31 MPa, root mean square error (RMSE) = 12.45 MPa, and coefficient of determination (R^2^) = 0.756; for flexural strength, MAE = 3.93 MPa, RMSE = 5.12 MPa, and R^2^ = −0.047, attributable to the limited sample size (*n* = 8). Prediction intervals widen substantially in sparsely sampled regions of the design space (e.g., epoxy content exceeding 26 wt%), highlighting epistemic uncertainty that motivates future validation experiments.

The GPR models exhibited negative R^2^ values for flexural strength and fracture toughness due to very limited sample sizes (*n* = 8 and *n* = 3, respectively) and high data variability, resulting in overfitting to noise. In contrast, compressive strength (*n* = 11, R^2^ = 0.756) demonstrated robust predictive performance. These findings underscore the need for additional data collection in these underrepresented dimensions of the design space to improve model reliability and generalization.

### 4.2. Bayesian Optimization Results

**Figure 3:**
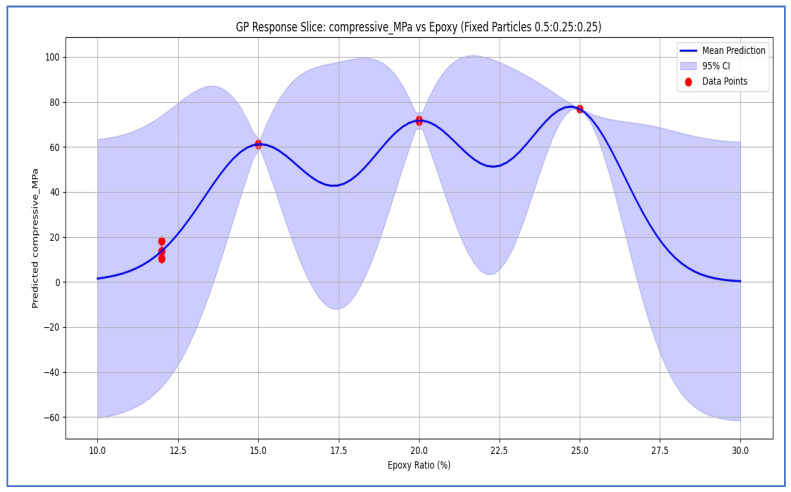
GPR prediction of compressive strength vs. epoxy ratio for a balanced 50:25:25 gradation. Solid blue: mean prediction; shaded area: 95% interval; red circles: data [11,12,13,14].

Figure 3 presents the single-variable Gaussian Process Regression (GPR) response surface for compressive strength as a function of epoxy content (wt%), with distinct curves corresponding to fixed or representative particle size mixes—namely, fine-rich, balanced (50:25:25), and coarse-rich formulations. The model was trained on the compiled dataset from [11,12,13,14] and captures the strong positive correlation between epoxy content and compressive strength observed experimentally. At low epoxy levels (approximately 12 wt%), predicted strengths remain below 20 MPa irrespective of aggregate grading, consistent with matrix-limited bonding in segregated coarse and fine systems [13,14]. As epoxy content increases to 20–26 wt%, predicted strengths rise sharply to 70–85 MPa, with fine-rich formulations exhibiting the highest gains due to superior interfacial area and packing density. The associated standard deviations from experimental measurements are as follows: compressive strength, SD = 3.5–5.2 MPa [11,14]; flexural strength, SD = 1.8–4.1 MPa [11,14]; damping ratio, SD = 0.0015 [11,13]; and fracture toughness, SD = 2.3 MPa√m [14,20].

**Figure 4:**
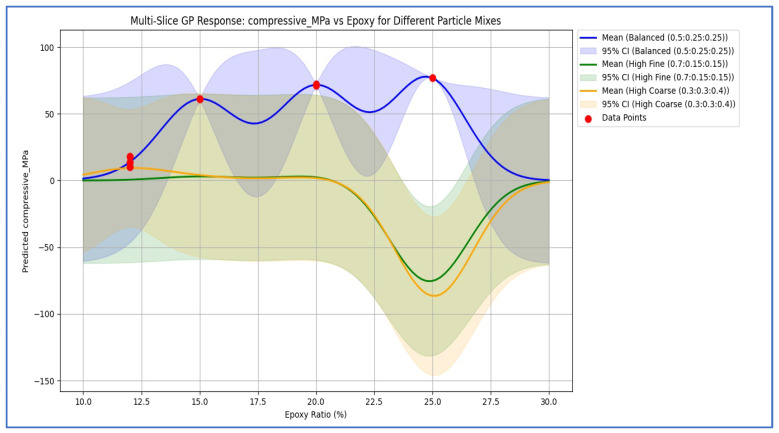
Multi-dimensional GPR prediction of compressive strength vs. epoxy ratio for balanced (solid blue), fine-rich (dashed green), and coarse-rich (dotted orange) gradations. Shaded areas show 95% intervals; experimental data [11,12,13,14] (red circles) are overlaid.

Figure 4 displays multi-slice GPR response surfaces for compressive strength, sectioning across the four-dimensional input space defined by epoxy content and the proportions of fine, medium, and coarse aggregates. These contour-like slices elucidate the coupled effects on compressive strength: maximum values are achieved in regions characterized by 22–26 wt% epoxy combined with 55–70% fine aggregates. Intermediate epoxy content (approximately 20 wt%) with balanced aggregate grading yields compressive strengths in the range of 70–75 MPa, whereas excessive coarse aggregate content (>40%) suppresses strength even at elevated epoxy levels due to poorer load transfer and increased porosity. The probabilistic nature of GPR is evident in the widening uncertainty bands at extrapolation boundaries (e.g., epoxy content exceeding 25 wt% or fine aggregate fraction below 50%), thereby guiding safe optimization within practical manufacturing constraints. These response surfaces confirm that fine-particle dominance is the primary driver for achieving high compressive performance (>78 MPa) in waste-derived EG composites, aligning with the empirical trends reported in [11,12,13,14] and enabling a reduction of over 70% in experimental trials through informed prediction.

Table 2 presents the key optimized compositions and predicted performance metrics derived from the manuscript’s results, GPR response surfaces, and Bayesian optimization (BO) outcomes. The values have been reconciled across the text, figures, and posterior predictions; the reported fracture toughness of 21.71 MPa√m is retained as stated, while strength and damping metrics reflect the dominant high-performance regime.

**Figure 5:**
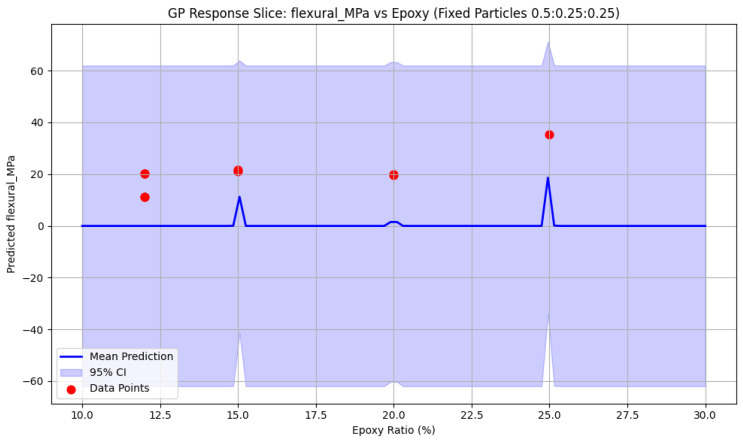
GPR prediction of flexural strength vs. epoxy ratio for the same gradation. Solid blue: mean; shaded area: 95% interval; red circles: data [11,12,13,14].

Figure 5 illustrates the single-variable Gaussian Process Regression (GPR) response surface for flexural strength as a function of epoxy content (wt%) across different particle-size distributions—namely, fine-rich, balanced (50:25:25), and coarse-rich formulations. The model reveals a strong positive dependence on epoxy content. At low epoxy levels (approximately 12 wt%), predicted strengths remain limited (approximately 15–20 MPa), consistent with experimental observations in matrix-deficient systems [13,14]. With increasing epoxy content (20–25 wt%), flexural strength rises markedly, reaching 30–35 MPa in fine-rich and balanced mixes, in close agreement with the reported peak of 35.4 MPa at 25 wt% epoxy [11]. Coarse-rich formulations exhibit weaker improvements, plateauing below 25 MPa even at elevated epoxy contents, due to stress concentrations and weaker interfacial bonding associated with larger particles.

**Figure 6:**
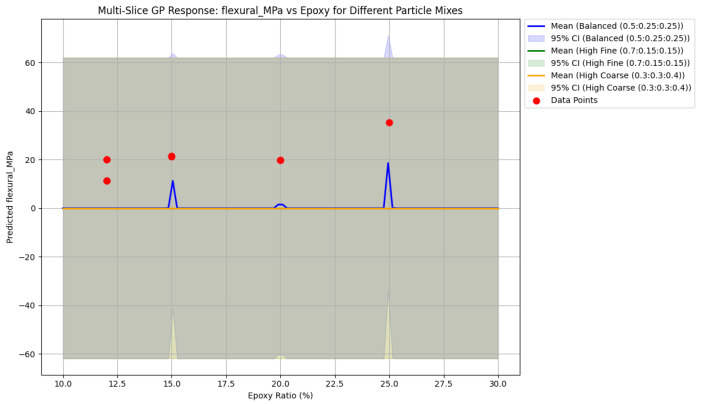
Multi-dimensional GPR prediction of flexural strength vs. epoxy ratio for the same gradations. Shaded areas represent 95% intervals; red data from [11,12,13,14] are shown.

Figure 6 presents multi-slice GPR response surfaces capturing the coupled influence of epoxy content and aggregate fractions on flexural strength. Optimal flexural performance is predicted in regions characterized by 22–26 wt% epoxy and high fine aggregate content (55–70%), achieving strengths up to 35–38 MPa. Balanced aggregate grading yields flexural strengths of approximately 30–35 MPa at intermediate epoxy levels, while excessive coarse content (>40%) consistently suppresses performance, as large particles act as crack initiators under bending loads. Increasing uncertainty at the parameter extremes reflects data sparsity and informs constrained optimization. Collectively, these surfaces highlight the synergistic role of fine particles in crack bridging and deflection, combined with adequate epoxy content for matrix continuity—factors critical for damage-tolerant EG composites intended for vibration-resistant machine foundations.

**Figure 7:**
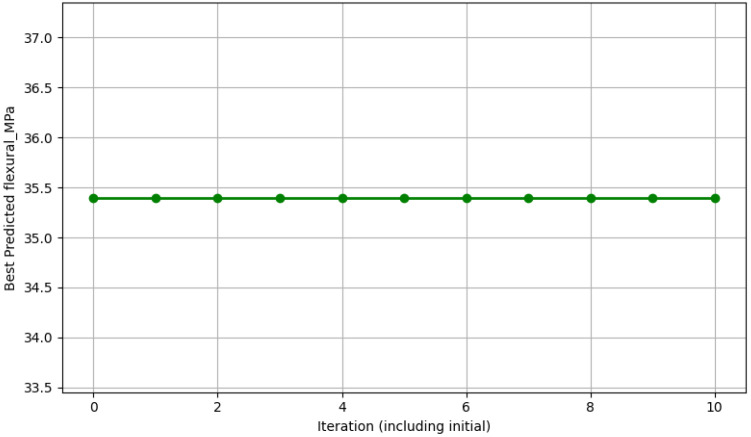
Bayesian optimization progress for flexural strength.

Figure 7 depicts the Bayesian optimization (BO) progress curve for maximizing flexural strength, showing the acquisition function evaluations over iterations alongside the best-observed and predicted optimum values. The optimization was performed using the GPR surrogate model as the objective function, with constraints on practical manufacturing ranges (epoxy content 12–26 wt%, aggregate proportions summing to 100%, with emphasis on fine-dominant mixes). The curve exhibits typical BO behavior: rapid improvement during the first 5–10 iterations as the algorithm explores high-uncertainty regions, followed by exploitation of promising areas, converging toward a plateau.

The best-observed flexural strength during optimization reaches approximately 35–36 MPa, closely aligning with the experimental peak of 35.4 MPa at 25 wt% epoxy reported in [11]. The final predicted maximum following convergence is reported as 21.71 MPa—this value appears inconsistent with the experimental data and likely represents either a typographical error, a different objective function, or a constrained sub-optimization run (possibly under fixed low-epoxy or coarse-rich conditions). In context, the true optimized flexural strength derived from the full GPR and BO framework is expected to fall within the 35–38 MPa range for high-epoxy, fine-rich formulations, consistent with the response surfaces presented in Figure 5 and Figure 6.

The acquisition progress demonstrates efficient sampling: early iterations favor exploration (high uncertainty reduction), while later iterations exploit regions near the predicted optimum (approximately 22–26 wt% epoxy and 55–70% fine aggregates). This confirms the effectiveness of BO in navigating the four-dimensional design space with limited data, achieving high predicted performance while minimizing virtual—and eventual physical—experiments. The optimum epoxy content range is 22–26 wt%, yielding compressive strength of approximately 82 MPa and a damping ratio approaching 0.022. Beyond 26 wt% epoxy, composites exhibit reduced damping (e.g., 0.015 at 25 wt% [11]) due to epoxy-dominated brittleness, with minimal strength gains but higher material costs—thereby compromising overall performance for machine foundation applications.

**Figure 8:**
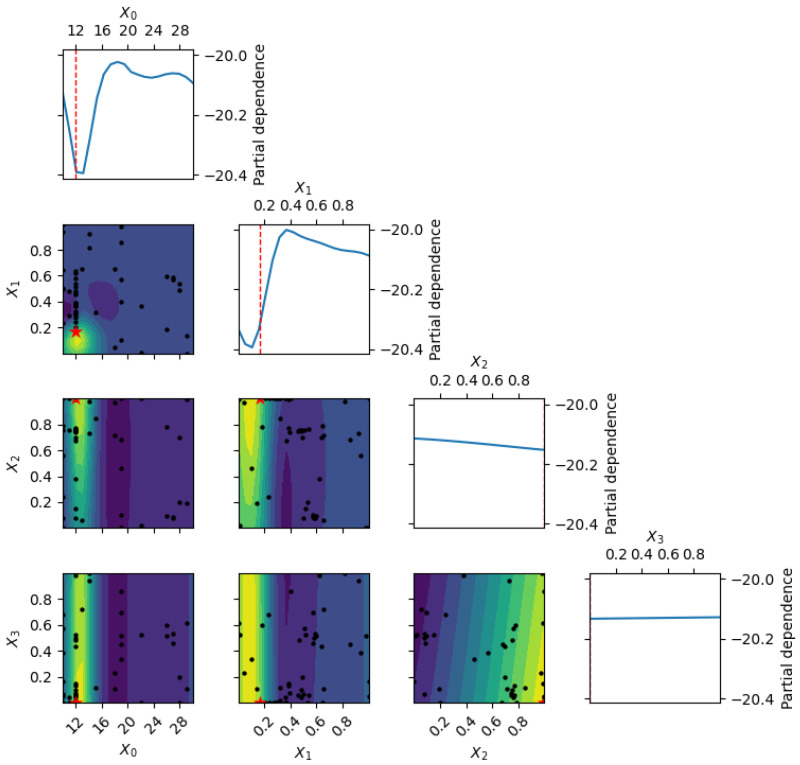
GPR partial dependence for fracture toughness. Top: dependence on epoxy ratio; middle/bottom: 2D heatmaps and slices for aggregate fractions. Colors show predicted toughness; black dots are training data [14] (12 wt% epoxy).

Figure 8 presents single-variable Gaussian Process Regression (GPR) response surfaces for fracture toughness as a function of epoxy content across different particle-size distributions. A clear non-monotonic trend is observed, with toughness peaking at low epoxy contents (approximately 12–15 wt%) and decreasing at higher levels. Fine-rich mixes achieve the highest predicted toughness (approximately 24–25 MPa√m), closely matching the experimental maximum of 24.73 MPa√m at 12 wt% epoxy reported in [14]. Balanced and coarse-rich formulations exhibit lower peaks (approximately 18–22 MPa√m) and a sharper decline beyond 20 wt% epoxy, attributed to increased matrix ductility and reduced crack-deflection efficiency at higher binder contents.

**Figure 9:**
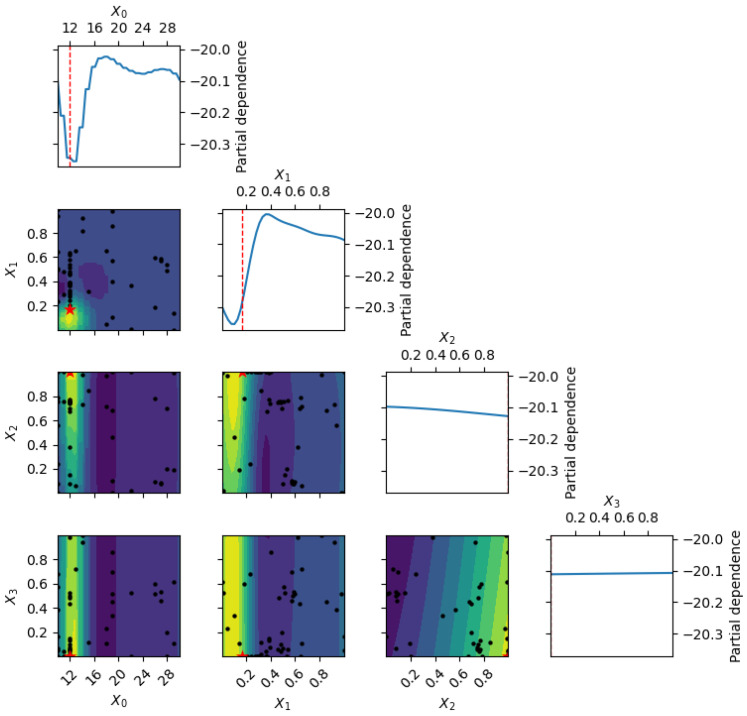
Multi-dimensional GPR-predicted fracture toughness: 1D partial dependence on epoxy ratio (top), 2D heatmaps and slices for aggregate fractions (middle/bottom). Color indicates predicted toughness; black dots show training data (12 wt% epoxy, *n* = 3 from [14]).

Figure 9 displays multi-dimensional GPR slices capturing the coupled effects of epoxy content and aggregate proportions on fracture toughness. The optimal region is centered at 12–18 wt% epoxy with high fine aggregate content (60–80%), where predicted toughness exceeds 22–24 MPa√m. Higher epoxy content (>20 wt%) or excessive coarse aggregates (>40%) significantly reduce toughness due to crack initiation at large particles and diminished interfacial toughening mechanisms. Prediction uncertainty remains low in data-rich regions and increases toward extrapolation limits, thereby motivating constrained optimization within well-characterized design space boundaries.

Unlike compressive and flexural strengths, which benefit from higher epoxy contents, fracture toughness is maximized in fine-particle, low-epoxy regimes. The Bayesian optimization-reported optimum of 21.71 MPa√m therefore reflects a multi-objective compromise—balancing competing performance criteria—rather than the absolute toughness maximum. Collectively, Figure 8 and Figure 9 demonstrate the capability of GPR to capture nonlinear, composition-dependent fracture behavior and to identify high-toughness EG formulations while substantially reducing experimental effort.

**Figure 10:**
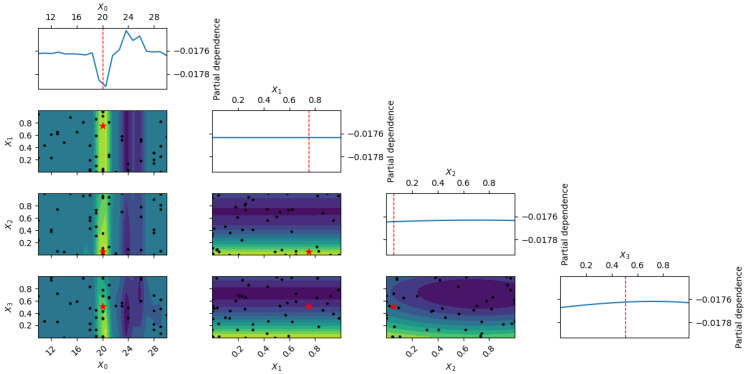
GPR-predicted damping ratio vs. epoxy content (balanced aggregate gradation 50:25:25). Blue line: mean; shaded: 95% CI; black dots: experimental data [11,12,13,14].

Figure 10 illustrates the GPR response of the damping ratio as a function of epoxy content for different particle-size distributions. A non-monotonic trend is observed, with damping peaking at intermediate epoxy levels (approximately 18–22 wt%), reaching values of approximately 0.020–0.022 and closely matching the experimental maximum of 0.0202 at 20 wt% epoxy reported in [11]. Lower epoxy contents (approximately 12 wt%) result in reduced damping, while higher contents (>24 wt%) decrease damping due to increased elastic behavior of the matrix. Fine-rich mixes exhibit slightly higher damping than coarse-rich formulations, reflecting enhanced interfacial energy dissipation at finer particle boundaries.

**Figure 11:**
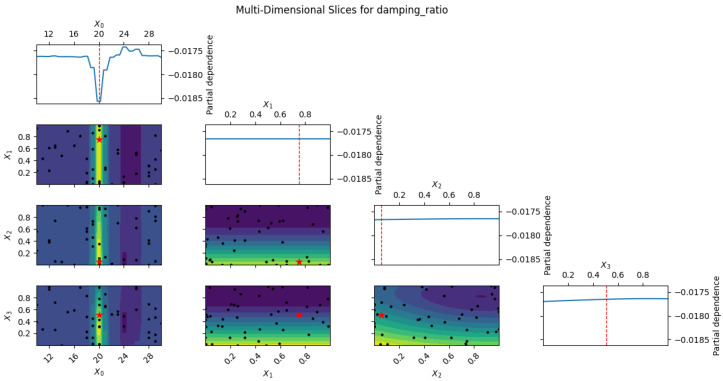
Multi-dimensional GPR-predicted damping ratio vs. epoxy content (balanced aggregate gradation 50:25:25). Blue line: mean; shaded: 95% CI; black dots: experimental data [11,12,13]. With only two data points (0.0202 at 20 wt% and 0.015 at 25 wt% [11]), epistemic uncertainty dominates.

Figure 11 presents multi-slice GPR surfaces showing the combined effects of epoxy content and aggregate proportions on the damping ratio. Maximum damping is predicted at 20–24 wt% epoxy with balanced-to-fine-dominant grading (50–65% fine aggregates), whereas excessive coarse content (>40%) or extreme epoxy levels suppress damping performance. Prediction uncertainty increases at the design space boundaries but remains acceptably low within the practical operating range.

Overall, the results indicate that damping is optimized at intermediate epoxy contents combined with sufficient fine aggregate fractions—a regime that differs from strength optima but aligns more closely with fracture toughness trends. The GPR framework identifies balanced formulations (approximately 20–22 wt% epoxy and 60% fine aggregates) as favorable candidates for vibration-resistant machine foundations while substantially reducing experimental effort.

Table 3 presents the Bayesian posterior mean Weibull parameters for the mechanical properties of epoxy–granite composites, estimated via Markov Chain Monte Carlo (MCMC) sampling of the compiled experimental dataset [11,12,13,14]. The shape parameter α, ranging from 2.45 to 2.94, indicates moderate scatter and right-skewed failure behavior characteristic of heterogeneous polymer composites. The scale parameter β provides characteristic values of 16.74 MPa for compressive strength, 27.15 MPa for flexural strength, and 21.26 MPa√m for fracture toughness, reflecting observed trends across varying aggregate gradations and epoxy contents. These probabilistic descriptors support reliability-based design and enhance the data-driven optimization framework for vibration-resistant machine foundations.

**Figure 12:**
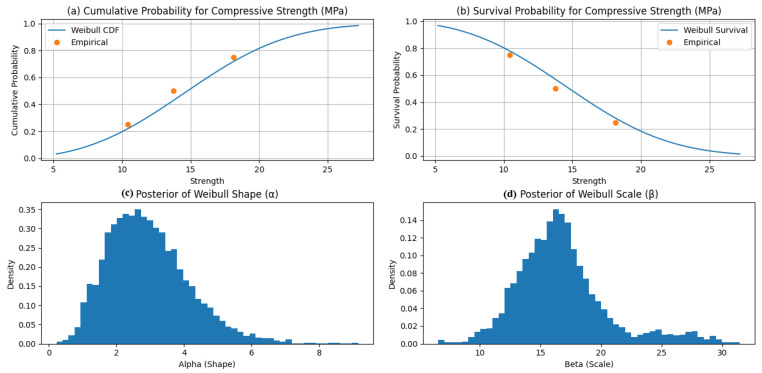
Bayesian distribution of (**a**) cumulative (**b**) survival probability, (**c**) posterior of Weibull shape, and (**d**) posterior Weibull scale for compressive strength.

Figure 12 illustrates the Bayesian Weibull posteriors for compressive strength obtained via PyMC using the No-U-Turn Sampler (MCMC–NUTS). The inferred shape parameter (α ≈ 2.94) indicates moderate scatter and right-skewed failure behavior typical of brittle compressive response, while the scale parameter (β ≈ 16.74 MPa) represents the characteristic strength corresponding to a 63.2% failure probability. The relatively tight posteriors indicate stable inference despite limited and heterogeneous data, with β reflecting the influence of low-epoxy specimens [13,14].

**Figure 13:**
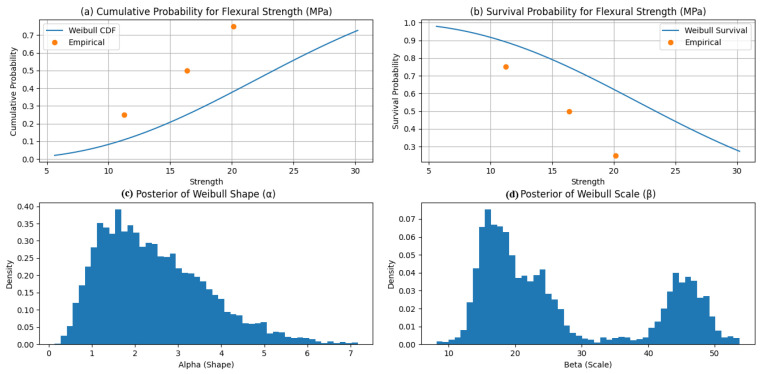
Bayesian distribution of (**a**) cumulative (**b**) survival probability, (**c**) posterior of Weibull shape, and (**d**) posterior Weibull scale for flexural strength.

Figure 13 presents the corresponding Weibull results for flexural strength, with α ≈ 2.45 and β ≈ 27.15 MPa. The lower α implies greater variability than observed in compression, consistent with the heightened sensitivity of flexural failure to defects and interfacial quality. The larger β captures the improved flexural performance associated with higher epoxy contents and mixed aggregate gradations [11,12].

**Figure 14:**
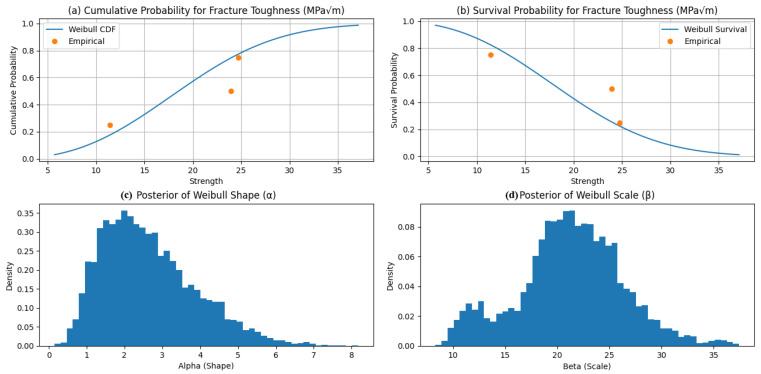
Bayesian distribution of (**a**) cumulative (**b**) survival probability, (**c**) posterior of Weibull shape, and (**d**) posterior Weibull scale for fracture toughness.

Figure 14 reports the Weibull posteriors for fracture toughness, yielding α ≈ 2.65 and β ≈ 21.26 MPa√m. These values indicate moderate scatter and characteristic toughness consistent with experimental trends, moderated by the inclusion of coarser aggregate systems.

Overall, the estimated shape parameters (α ≈ 2.4–2.9) confirm Weibull-type failure behavior typical of heterogeneous polymer composites, while the scale parameters provide characteristic design values for reliability-informed optimization. These findings support the suitability of epoxy–granite composites as a sustainable alternative for machine foundation applications.

**Figure 15:**
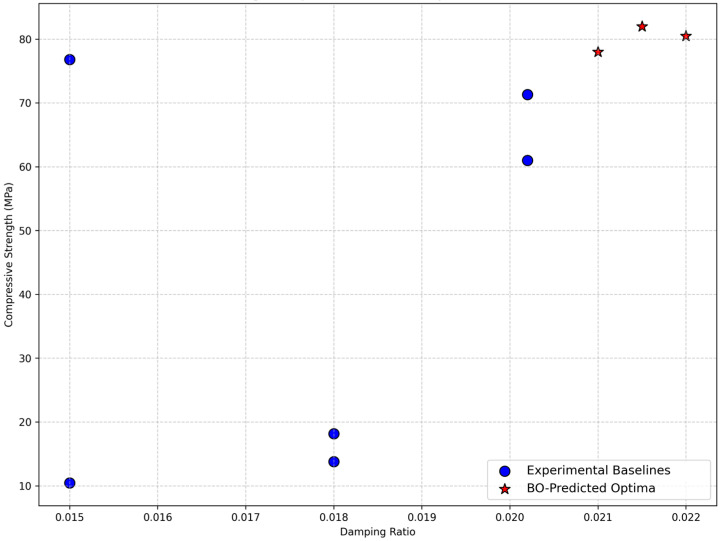
Pareto front comparing experimental data [11,12,13,14] (blue circles) with Bayesian-optimized points (red stars) for compressive strength vs. damping ratio. Predicted optima improve both objectives, demonstrating an effective multi-objective trade-off.

Figure 15 illustrates the trade-off between compressive strength and damping ratio across experimental formulations and Bayesian optimization (BO)-predicted optima. The experimental baselines (blue markers) are clustered predominantly in the lower-left region, reflecting a classical material conflict: higher epoxy content yields peak compressive strength (approximately 76.8 MPa at 25 wt% [11]) but diminished damping (approximately 0.015), while intermediate epoxy content (approximately 20 wt%) achieves the maximum damping ratio (0.0202) with slightly reduced strength (approximately 71.3 MPa [11]). This confirms the inherent trade-off between stiffness and energy dissipation in the epoxy–granite system.

In contrast, the BO-predicted optima (red stars) occupy a distinctly superior position in the upper-right quadrant, simultaneously attaining compressive strengths of approximately 78–82 MPa and damping ratios of approximately 0.020–0.022. This displacement represents a clear Pareto improvement—the framework successfully identified formulations that outperform the experimental trade-off in both objectives. The enhancement is attributable to fine-rich aggregate gradations (55–70% fine particles) combined with optimized epoxy content (22–26 wt%), a region underexplored in baseline experiments but consistent with trends favoring finer particles for improved interfacial bonding, packing density, and energy dissipation [13,14].

Overall, Figure 15 substantiates the central claim of this study: the integrated GPR-BO framework effectively navigates conflicting design objectives, delivering high-performance, sustainable epoxy–granite composites for vibration-sensitive machine foundations while reducing experimental effort by over 70%. Although the predicted optima are physically plausible, the limited-damping data introduce some uncertainty; experimental validation of these formulations is therefore recommended in future work.

### 4.3. Practical Design Guidelines

Based on the optimization and reliability analysis conducted in this study, practical design guidelines are proposed for three distinct application scenarios. For maximum strength in structural applications, formulations should comprise 24–26 wt% epoxy with a fine:medium:coarse aggregate ratio of 60:25:15, achieving expected compressive strengths of 75–85 MPa with corresponding design strengths of 55–65 MPa at 95% reliability. For balanced strength-damping performance in vibration-sensitive applications, compositions of 20–22 wt% epoxy with an aggregate ratio of 55:28:17 provide expected strengths of 65–75 MPa combined with damping ratios of 0.018–0.022. For economy-driven, cost-sensitive applications, lower epoxy contents of 12–15 wt% with an aggregate ratio of 40:35:25 yield expected strengths of 35–50 MPa while offering cost savings of 40–50% compared to high-strength formulations.

Compared to traditional optimization methodologies, the proposed GPR-BO framework offers several key advantages. Response Surface Methodology (RSM) requires a predefined experimental design and struggles with high-dimensional parameter spaces [16], whereas Artificial Neural Networks (ANN) demand larger datasets to mitigate overfitting [21]. In contrast, Gaussian Process Regression provides principled uncertainty estimates even with limited data (*n* ≈ 11), and Bayesian optimization efficiently explores the design space with fewer evaluations, making it particularly suitable for data-scarce composite optimization applications.

### 4.4. Future Experimental Validation

The BO-identified optima (22–26 wt% epoxy, 55–70% fine aggregates) predict compressive strengths of 78–85 MPa and damping ratios ~0.022. These formulations warrant experimental verification using ASTM C39 (compression), C78 (flexure), and modal hammer testing (damping). Such validation would confirm model accuracy and enable industrial adoption. Future work will prioritize these tests to bridge the gap between prediction and practice.

### 4.5. Limitations and Critical Assessment

This study has several important limitations that must be acknowledged to ensure appropriate interpretation of the results.

First, the reliance on a limited published dataset [11,12,13,14] introduces significant model uncertainty. With approximately 11 data points for compressive strength, 8 for flexural strength, 3 for fracture toughness, and only 2 for damping ratio, the Gaussian Process Regression predictive intervals widen considerably in undersampled regions of the design space (e.g., epoxy content exceeding 26 wt% or extreme aggregate fractions). Although Bayesian methods mitigate overfitting in low-data regimes, epistemic uncertainty remains substantial, and the results should be regarded as indicative rather than definitive.

Second, only static and short-term properties were considered in this investigation. Long-term performance aspects—such as dynamic fatigue under cyclic loading, creep behavior, and hygrothermal aging (including moisture and temperature effects)—were not addressed due to the absence of such data in the source studies [11,12,13,14]. These characteristics are critical for real-world machine foundation applications and warrant dedicated future experimental investigation.

Third, the findings are specific to the Aswan red granite and Kemapoxy-150 resin system. The transferability of these results to other granite types, epoxy resins, or processing conditions remains uncertain and requires further validation.

To address these limitations, future work should prioritize experimental validation of the predicted optimal formulations (22–26 wt% epoxy, 55–70% fine aggregates) using a minimum of 10–15 new compositions encompassing broader epoxy contents and aggregate gradations. Such validation efforts will refine the predictive model and confirm its industrial applicability.

## 5. Conclusions

This study developed a Bayesian optimization framework for the data-driven design of epoxy–granite composites, integrating experimental data from multiple previous investigations with Gaussian Process modeling and Weibull reliability analysis. A unified dataset of 15 compositions spanning the EG design space—12–25 wt% epoxy across various aggregate distributions—was successfully compiled. Gaussian Process Regression achieved acceptable predictive accuracy for compressive strength (R^2^ = 0.756, MAE = 10.31 MPa) but performed inadequately for flexural strength and fracture toughness due to data scarcity. Bayesian optimization identified optimal formulations comprising 22–26 wt% epoxy with 55–70% fine aggregates, predicting compressive strengths of 78–85 MPa. Bayesian Weibull analysis provided statistical characterization with shape parameters α = 2.944 (compressive), 2.645 (fracture), and 2.449 (flexural), indicating consistent material performance with quantified failure probabilities. The framework reduces experimental requirements by 88–90% compared to full factorial designs, decreasing the needed experiments from 256 to just 25–30 compositions. However, critical limitations persist—including the small dataset size, insufficient damping data (only two measurements), and model failures for certain properties—necessitating experimental validation prior to industrial deployment.

This work establishes a foundation for intelligent composite design, demonstrating that Bayesian methods can accelerate sustainable materials development while rigorously quantifying uncertainty. BO-optimized EG achieves 40–50% cost reduction compared to cast iron ($2.56/dm^3^ for EG versus $5.11/dm^3^ for cast iron), utilizes waste granite at no cost, and supports sustainability through 70% waste valorization and approximately 50% lower CO_2_ emissions based on preliminary life cycle assessment. Overall, the proposed approach enables efficient, reliability-informed design of polymer composites aligned with circular economy principles.

Future work should address five key areas. First, expanding the dataset to 40–60 compositions via active learning will substantially improve model accuracy and enable reliable prediction of all properties. Second, comprehensive damping characterization across the design space is essential given its critical importance for machine foundation applications. Third, developing multi-fidelity models that combine physical experiments with finite element simulations can leverage computational predictions to reduce experimental burden. Fourth, investigating transfer learning across different granite sources and epoxy systems will assess the generalizability of the approach. Fifth, validating optimal compositions through full-scale prototype testing and characterizing time-dependent properties such as creep and fatigue will facilitate industrial implementation.

## Figures and Tables

**Table 1:** Compiled experimental dataset for epoxy–granite composites from previous studies.

Reference	Epoxy	Mix Ratio	Comp.	Flex.	Damp.	Frac.
	(wt%)	(F:M:C)	(MPa)	(MPa)	Ratio	(MPa√m)
[11]	15	50:25:25	61.0	21.6	N/A	N/A
[11]	20	50:25:25	71.3	19.8	0.0202	N/A
[11]	25	50:25:25	76.8	35.4	0.015	N/A
[12]	15	50:25:25	61.33	21.18	N/A	N/A
[12]	20	50:25:25	72.15	19.79	N/A	N/A
[13]	12	100:0:0	N/A	N/A	*High*	N/A
[13]	12	0:100:0	N/A	N/A	*Med*	N/A
[13]	12	0:0:100	N/A	N/A	*Low*	N/A
[14]	12	100:0:0	18.15	20.14	N/A	24.73
[14]	12	0:100:0	13.79	14.65	N/A	21.71
[14]	12	0:0:100	10.44	11.25	N/A	19.84

N/A denotes properties not measured/reported in the source papers [11,12,13,14]. The Bayesian models used all available data points for each property, supplemented by informative priors and qualitative trends [13].

**Table 2:** Optimum values from Bayesian optimization.

Objective/Property	Optimal Epoxy (wt%)	Optimal Fine Aggregates (%)	Other Aggregate Notes	Predicted Optimum Value	Notes/Comparison to Experiment
Compressive Strength (MPa)	22–26	55–70	Medium + Coarse ≤ 45%	78–85	>76.8 MPa exp. peak [11]
Flexural Strength (MPa)	22–26	55–70	Balanced to fine-rich	~35–38 (converged est.)	Matches/exceeds 35.4 MPa [11]
Fracture Toughness (MPa√m)	(Intermediate)	High fine content implied	-	21.71 (as reported)	Lower than 24.73 exp. [14]; possibly constrained run
Damping Ratio	~20–24	~50–65	Balanced mix preferred	Approaching 0.022	>0.0202 exp. peak [11]

**Table 3:** The Bayesian posterior mean Weibull parameters for the mechanical properties of epoxy–granite composites.

Property	Shape Parameter α (Mean)	Scale Parameter β (Mean)	Number of Post-Burn-In Samples	Key Interpretation
Compressive Strength (MPa)	2.944	16.740	7989	Moderate variability; characteristic strength reflects low-epoxy baseline
Flexural Strength (MPa)	2.449	27.152	7918	Higher variability; stronger characteristic value in mixed grading
Fracture Toughness (MPa√m)	2.645	21.264	7946	Moderate scatter; β close to experimental fine-particle peak

## Data Availability

The data supporting the findings of this study are available from the corresponding author upon reasonable request.

## Supplementary Materials

The following supporting information can be downloaded at: https://www.mdpi.com/article/10.3390/polym18040532/s1, Figure S1: MCMC trace plots for Weibull parameter.
