# Supplementary material for: Data-Driven Design of Epoxy–Granite Machine Foundations: Bayesian Optimization for Enhanced Compressive Strength and Vibration Damping
Source: Polymers (Basel). 2026 Feb 21;18(4):532. doi: 10.3390/polym18040532 (PMC12944673; doi:10.3390/polym18040532)
Supplement: Supplementary file 1 [file polymers-18-00532-s001.zip › polymers-4157335-supplementary.pdf]

Article

Data-Driven Design of Epoxy–Granite Machine Foundations: Bayesian Optimization for Enhanced Compressive Strength and Vibration Damping

Mohammed Y. Abdellah [1,2,*], Osama M. Irfan [3,4,*] and Hanafy M. Omar [3]

[1] Mechanical Engineering Department, Faculty of Engineering, Qena University, Qena 83521, Egypt
[2] Mechanical Engineering Department, College of Engineering, Alasala, Dammam 31483, Saudi Arabia
[3] Department of Mechanical Engineering, College of Engineering, Qassim University, Buraydah 51452, Saudi Arabia; hanafy@qu.edu.sa
[4] Department of Production Engineering, Beni Suef University, Beni Suef 62521, Egypt
* Correspondence: mohamed_abdalla@eng.svu.edu.eg (M.Y.A.); o.ahmed@qu.edu.sa (O.M.I.)

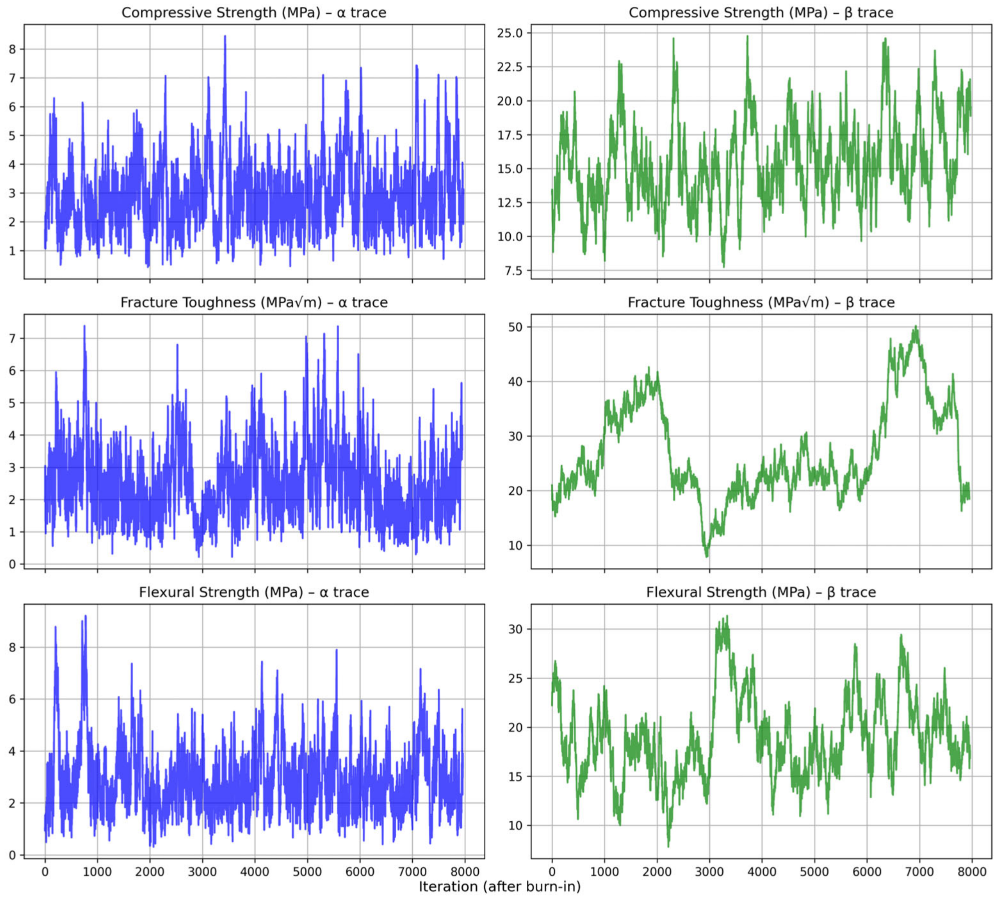

Figure S1. MCMC trace plots for Weibull parameter.

Academic Editor: Vagelis Harmandaris

Received: 31 January 2026
Revised: 14 February 2026
Accepted: 19 February 2026
Published: date

Copyright: © 2026 by the authors. Submitted for possible open access publication under the terms and conditions of the Creative Commons Attribution (CC BY) license.
